# Discovery of Novel Natural Inhibitors of H5N1 Neuraminidase Using Integrated Molecular Modeling and ADMET Prediction

**DOI:** 10.3390/bioengineering12060622

**Published:** 2025-06-07

**Authors:** Afaf Zekri, Mebarka Ouassaf, Shafi Ullah Khan, Kannan R. R. Rengasamy, Bader Y. Alhatlani

**Affiliations:** 1 Group of Computational and Medicinal Chemistry, LMCE Laboratory, University of Biskra, BP 145, Biskra 07000, Algeria; afaf.zekri@univ-biskra.dz; 2 Inserm U1086 ANTICIPE (Interdisciplinary Research Unit for Cancer Prevention and Treatment), Normandie University, Université de Caen Normandie, 14076 Caen, France; shafiullahpharmd@gmail.com; 3 Comprehensive Cancer Center François Baclesse, UNICANCER, 14076 Caen, France; 4 Laboratory of Natural Products and Medicinal Chemistry (LNPMC), Saveetha Medicinal College and Hospitals, Saveetha Institute of Medical and Technical Sciences (SIMATS), Thandalam, Chennai 602105, India; kannan@LNPMC.in; 5 Centre of Excellence for Pharmaceutical Sciences, North-West University, Potchefstroom 2520, South Africa; 6 Unit of Scientific Research, Applied College, Qassim University, Buraydah 52571, Saudi Arabia

**Keywords:** ADMET, molecular docking, molecular dynamics simulations, neuraminidase inhibitor, Zanamivir

## Abstract

The avian influenza virus, particularly the highly pathogenic H5N1 subtype, represents a significant public health threat due to its interspecies transmission potential and growing resistance to current antiviral therapies. To address this, the identification of novel and effective neuraminidase (NA) inhibitors is critical. In this study, an integrated in silico strategy was employed, beginning with the generation of an energy-optimized pharmacophore model (e-pharmacophore, ADDN) based on the reference inhibitor Zanamivir. A virtual screening of 47,781 natural compounds from the PubChem database was performed, followed by molecular docking validated through an enrichment assay. Promising hits were further evaluated via ADMET predictions, density functional theory (DFT) calculations to assess chemical reactivity, and molecular dynamics (MD) simulations to examine the stability of the ligand–protein complexes. Three lead compounds (C1: CID 102209473, C2: CID 85692821, and C3: CID 45379525) demonstrated strong binding affinity toward NA. Their ADMET profiles predicted favorable bioavailability and low toxicity. The DFT analyses indicated suitable chemical reactivity, particularly for C2 and C3. The MD simulations confirmed the structural stability of all three ligand–NA complexes, supported by robust and complementary intermolecular interactions. In contrast, Zanamivir exhibited limited hydrophobic interactions, compromising its binding stability within the active site. These findings offer a rational foundation for further experimental validation and the development of next-generation NA inhibitors derived from natural sources.

## 1. Introduction

The avian influenza virus is highly transmissible and responsible for acute respiratory infections that range in severity from mild symptoms—similar to seasonal influenza—to life-threatening complications, including severe pneumonia and multi-organ failure [1]. Among the four known types of influenza viruses, Influenza A is the most virulent, playing a central role in both epidemics and pandemics due to its zoonotic potential and capacity for genetic reassortment [2,3]. This virus infects a wide spectrum of hosts, including numerous avian species and several mammalian ones, such as humans, pigs, and horses.

**Figure 1:**
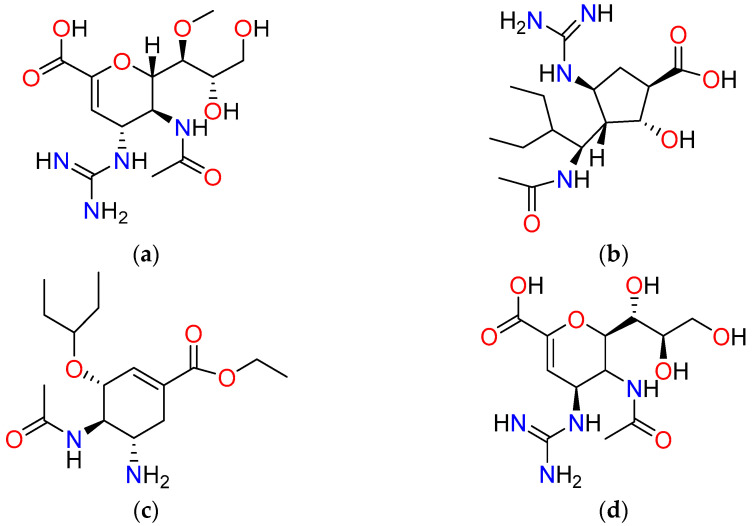
Illustration of 2D structures of compounds known to be neuraminidase inhibitors: (**a**) Laninamivir; (**b**) Peramivir; (**c**) Oseltamivir; (**d**) Laninamivir.

Influenza A is defined by two major surface glycoproteins: hemagglutinin (HA) and neuraminidase (NA). HA is critical for the initial stage of infection, mediating the recognition and binding of sialic acid residues on the surface of host epithelial cells. Following viral replication, NA cleaves sialic acid from cellular glycoproteins and progeny virions, promoting the release and spread of new viral particles to adjacent cells [4]. Among its subtypes, H5N1 is recognized as one of the most pathogenic strains of Influenza A, with a high case fatality rate and significant zoonotic potential, making it a major public health concern [5]. Neuraminidase remains a validated antiviral target and has been the focus of therapeutic intervention due to its essential role in viral propagation. Approved neuraminidase inhibitors, such as Laninamivir, Peramivir, Oseltamivir, and Zanamivir (Figure 1), have shown efficacy in treating influenza infections [6,7,8,9]. However, the continual emergence of resistant viral variants highlights the urgent need to discover novel and more effective neuraminidase inhibitors.

Several in silico studies have investigated the molecular mechanisms of the H5N1 subtype of the Influenza A virus using a wide range of computational techniques [10,11,12,13]. In recent years, significant advancements have been made in understanding this virus, through both experimental research [14,15,16,17] and theoretical modeling approaches [11,18,19,20,21,22,23,24,25,26,27]. These complementary strategies have deepened insights into viral structure, function, and interaction with potential therapeutic agents. The integration of molecular modeling techniques such as two-dimensional and three-dimensional quantitative structure–activity relationship analyses (2D-QSAR and 3D-QSAR), molecular docking, and absorption, distribution, metabolism, excretion, and toxicity (ADMET) prediction has become a cornerstone of modern pharmaceutical research. These in silico approaches significantly accelerate the drug discovery process by reducing reliance on costly and time-consuming experimental synthesis and testing. Among these tools, molecular docking is particularly valuable for predicting the specific interactions between a ligand and the active site of a protein, thereby offering critical insights for the rational design and optimization of bioactive compounds [28,29].

In this study, an integrated computational approach was employed to facilitate the virtual screening, identification, and evaluation of novel neuraminidase inhibitors derived from natural sources. The neuraminidase enzyme from the H5N1 influenza strain—an attractive target for antiviral drug development—was selected as the molecular target. Its three-dimensional structure (PDB ID: 2HTQ) was retrieved from the Protein Data Bank and used in docking simulations. Key active site residues—ARG118, ASP151, ARG152, TRP178, GLU276, TYR347, and ARG371—were specifically analyzed due to their essential role in ligand binding and catalysis [4]. Accurate characterization of this binding pocket is crucial for structure-based drug design and for future applications such as site-directed mutagenesis studies [30]. To effectively target H5N1 neuraminidase, a structure-based virtual screening (SBVS) pipeline was implemented, integrating e-pharmacophore modeling and molecular docking. The pharmacophore model was generated based on the known inhibitor Zanamivir, defining critical features necessary for neuraminidase inhibition. This model guided the virtual screening of 47,781 natural compounds sourced from the PubChem database. Top-scoring hits were further evaluated through ADMET predictions to assess their pharmacokinetic behavior, safety profiles, and drug-likeness. This study’s innovation lies in its comprehensive and layered approach, integrating e-pharmacophore modeling, docking, ADMET predictions, density functional theory (DFT) analyses, and molecular dynamics (MD) simulations. Unlike previous studies that focused primarily on synthetic inhibitors or limited docking approaches, our methodology combines electronic property calculations and dynamic stability assessments to ensure both binding efficacy and adaptability in the biological context. This multifaceted strategy addresses the growing challenge of viral resistance and provides a robust framework for the discovery of potent, naturally derived neuraminidase inhibitors. The most promising compounds were further analyzed through DFT analyses to gain deeper insights into their electronic properties and chemical reactivity, while MD simulations were conducted to evaluate the dynamic stability and structural integrity of the top ligand–neuraminidase complexes over time.

## 2. Materials and Methods

### 2.1. Protein Preparation

The crystal structure of the NA protein from the avian influenza virus (PDB ID: 2HTQ), co-crystallized with the antiviral drug Zanamivir, was retrieved from the RCSB Protein Data Bank “http://www.rcsb.org/pdb (accessed on 8 February 2025)” [4]. The structure has a resolution of 2.20 Å, which falls within the optimal range of 1.5–2.5 Å, indicating high structural quality and suitability for molecular modeling [31,32]. Protein preparation was conducted using Maestro 11.8 (Schrödinger, 2018) with the OPLS4 force field, employing a simulation pH of 7.4 determined by PROPKA. The preparation process included filling missing side chains, optimizing hydrogen bond assignments, and deleting non-essential water molecules distant more than 5 Å from ligands. All small molecules (hets), including detected ligands, metals, ions, non-water solvents, and others, were removed to focus solely on the protein’s structure. Sample water orientations were optimized, and restrained minimization was applied until the heavy-atom RMSD reached 0.30 Å, ensuring structural stability. These parameters reflect rigorous settings that enhance the realism and reproducibility of the prepared model [33,34].

### 2.2. Pharmacophore Model Generation

The energy-optimized e-pharmacophore model was developed using the crystal structure of the NA protein (PDB ID: 2HTQ). Zanamivir, the co-crystallized ligand, was redocked into the protein’s active site using the Glide docking algorithm in Extra Precision (XP) mode to generate an accurate binding pose. Based on this protein–ligand complex, the e-pharmacophore model was constructed using the Phase module of the Schrödinger software suite, version 2018. The pharmacophore hypothesis was generated using Phase with the following settings: a maximum of five pharmacophoric features were allowed, and hydrogen bond donors were treated as vectors to account for directional interactions. The minimum distance between different features was set at 2.00 Å, while the minimum distance between features of the same type was set at 4.00 Å to avoid redundancy. Six key pharmacophoric features were identified in the model: hydrogen bond acceptor (A), hydrogen bond donor (D), hydrophobic region (H), negatively charged group (N), positively charged group (P), and aromatic ring (R). The resulting pharmacophore hypothesis was subsequently used to screen a library of natural compounds extracted from the PubChem database for potential NA inhibitors.

The pharmacophore model was generated based on the re-docked pose of Zanamivir, ensuring biological relevance and structural consistency. The feasibility and repeatability of pharmacophore generation were supported by employing standardized procedures in the Phase module of Schrödinger, with all parameters (feature definitions, maximum number of features, and distance constraints) clearly documented. This approach enables reproducibility and confirms the reliability of the model in identifying potential inhibitors.

### 2.3. Virtual Screening of PubChem Database

A virtual screening approach was employed to identify potential inhibitors of the H5N1 avian influenza virus by exploring a diverse set of natural compounds. A total of 47,781 natural compounds were initially retrieved from the PubChem database and subjected to preliminary filtering based on physicochemical properties using Canvas (Schrödinger). The applied selection criteria included molecular weight (MW) ≤ 400 Da, number of rotatable bonds (Rotatable B) ≤ 10, number of hydrogen bond donors (HBD) ≤ 5, number of hydrogen bond acceptors (HBA) ≤ 10, and octanol–water partition coefficient (XlogP) ≤ 5. Following this filtration process, 1829 compounds demonstrated a good pharmacophoric fit with a fitness score ≥ 1.5 and were subsequently selected for further molecular docking studies.

### 2.4. Molecular Docking and Enrichment Calculation

Molecular docking was performed using the Glide module from Schrödinger Suite [33]. The grid was generated by centering on the active site of the NA protein based on the bound position of the co-crystallized ligand Zanamivir [4]. Docking simulations were conducted in both Standard Precision (SP) and Extra Precision (XP) modes for compounds with fitness scores ≥ 1.5, ensuring a rigorous assessment of binding affinity. Ligand sampling was set to flexible, with enabled sampling of nitrogen inversions, ring conformations, and torsion sampling for all predefined functional groups, with penalties applied for nonplanar amide conformations. Epik state penalties were incorporated into the docking scores, van der Waals radii were scaled by a factor of 0.80, and a partial charge cutoff of 0.15 was used. Ligands exceeding 500 atoms or 100 rotatable bonds were excluded from the workflow to ensure computational feasibility. The accuracy of the docking protocol was evaluated through re-docking experiments, where Zanamivir and the well-known NA inhibitor Oseltamivir were docked back into the active site of the NA protein [35]. The resulting molecular poses were visualized using BIOVIA Discovery Studio 2016 [36], allowing for a detailed examination of ligand–protein interactions.

To validate the reliability of the docking procedure, an enrichment study was conducted using Schrödinger software. A Receiver Operating Characteristic (ROC) curve was generated by plotting sensitivity (true positives) against 1-specificity (false positives) [35,36]. Sensitivity reflects the docking algorithm’s ability to correctly identify 28 known active ligands, while specificity was assessed using the 28 active ligands with a decoy set of 1000 molecules, structurally similar to Zanamivir but presumed inactive, obtained from the PubChem database [37]. These compounds were docked using the SP mode of Glide, and the performance of the screening method was further assessed using the Robust Initial Enrichment (RIE) index [38,39]. The combination of ROC and RIE analyses confirmed the high reliability and predictive power of the molecular docking protocol.

An enrichment study was employed to validate the robustness and reliability of the virtual screening protocol. The use of a standardized set of active ligands and decoys, along with well-documented parameters and statistical evaluation (ROC and RIE), ensured the repeatability and reproducibility of the enrichment analysis.

### 2.5. Pharmacokinetic Properties and Drug-likeness

The absorption, distribution, metabolism, excretion, and toxicity (ADMET) characteristics of the most promising compounds identified from the molecular docking analysis were assessed using two web-based platforms. ADMETlab 3.0 (https://admetlab3.scbdd.com/, accessed on 10 February 2025) was used for detailed pharmacokinetic profiling, including properties related to oral bioavailability, metabolic stability, and excretory behavior. In parallel, the ProTox-3 web server (https://tox.charite.de/protox3/, accessed on 10 February 2025) was employed to estimate toxicity parameters, including hepatotoxicity and organ-specific toxicities. Together, these platforms provided comprehensive and reliable in silico predictions of pharmacokinetic and toxicological behavior, facilitating early-stage evaluation of the drug-likeness and safety of candidate compounds.

### 2.6. Density Functional Theory

The geometrical structures of the most promising compounds, identified by the molecular docking study, were optimized and analyzed using density functional theory (DFT). Calculations were performed using Gaussian 09 software [40] after pre-optimizing the three-dimensional structures with the molecular mechanics force field (MMFF+) and the semi-empirical Austin Model 1 (AM1) method [41] via HyperChem 8.0.8 software [42,43]. Geometry optimization and convergence were achieved using the BLYP functional with the 6-31G basis set [44], employing default Gaussian convergence criteria (energy change threshold of 10^−6^ Hartree; maximum force of 0.00045 Hartree/Bohr). All calculations were conducted in the gas phase, with electronic charge and multiplicity set to reflect the neutral ground state of the ligands. Vibrational frequency analysis was performed to confirm the absence of imaginary frequencies, validating the structural stability of the selected compounds (C1, C2, and C3). Subsequently, molecular orbital energies (HOMO and LUMO) and related global reactivity descriptors were calculated based on DFT results.

The resulting outputs were analyzed using the Gauss Sum program [45]. Various global reactivity descriptors were calculated from the energies of the highest occupied molecular orbital (HOMO) and the lowest unoccupied molecular orbital (LUMO) [46,47]. These descriptors include

(1) $${∆E}_{Gap}=E_{\mathrm{LUMO}}-E_{\mathrm{HOMO}},$$

η = (E_LUMO_ − E_HOMO_)/2, (2)

S =1/(2 η),(3)

μ = (E_LUMO_ + E_HOMO_)/2, (4)

χ = − (E_LUMO_ + E_HOMO_)/2, (5)

ω = µ^2^/2η, (6)

N = E_HOMO_ (Nucleophile) − E_HOMO_ (TCE), (7)

where ${∆E}_{Gap}$ is the energy gap, η is the chemical hardness, S is the chemical softness, μ is the chemical potential, χ is the electronegativity, ω is the electrophilicity index, and N is the nucleophilicity. These parameters allow for a thorough characterization of the electronic and reactive properties of the compounds studied.

The DFT calculations were carried out using Gaussian 09 with clearly specified parameters (functional, basis set, and convergence criteria), enabling reproducibility of geometry optimizations and ensuring the reliability of the computed electronic descriptors.

### 2.7. Molecular Dynamics Simulation

Molecular dynamics (MD) simulations were conducted using the Desmond software package from Schrödinger LLC (New York, NY, USA), version 2018 [33]. The simulations were performed under the NPT ensemble, with the temperature set at 300 K and pressure maintained at 1 bar [48]. Thermodynamic control was achieved using the Martyna–Tuckerman–Klein (MTK) chain coupling method for pressure (2.0 ps coupling constant) and the Nosé–Hoover chain thermostat for temperature regulation [49]. The OPLS_2005 force field was applied to accurately model molecular interactions [50]. Long-range electrostatics were treated with the Particle Mesh Ewald (PME) method and a 9.0 Å cutoff for Coulomb interactions [50]. Explicit solvation was modeled using the Simple Point Charge (SPC) water model [51], with Na^+^ and Cl^−^ counterions added to neutralize the simulation box and an ionic strength of 0.15 M applied to mimic physiological conditions. A cubic simulation box with a 10 Å buffer distance was constructed, and all bonds involving hydrogen atoms were constrained using the SHAKE algorithm to allow a 2 fs time step. Equilibration was conducted in two stages: 1 ns under the NVT ensemble for temperature stabilization, followed by 2 ns under the NPT ensemble for pressure equilibration prior to the 100 ns production simulation. Non-bonded interactions were calculated by updating short-range forces every step and long-range forces every three steps [52]. Trajectories were saved every 10 ps for analysis, and key dynamic parameters such as root mean square deviation (RMSD), root mean square fluctuation (RMSF), and the protein–ligand contact profile were monitored using the Simulation Interaction Diagram (SID) tool in Desmond [33]. The molecular dynamics simulations were conducted under standardized conditions using the Desmond software, version 2018, with detailed documentation of parameters such as force fields, temperature and pressure controls, and solvation protocols, ensuring the repeatability and reliability of the dynamic analysis.

Statistical analyses were incorporated into this study to ensure the robustness and validity of the virtual screening and molecular docking methodologies. The performance of the docking protocol was assessed by generating a Receiver Operating Characteristic (ROC) curve, calculating the area under the curve (AUC) to measure sensitivity and specificity [35,36]. The Robust Initial Enrichment (RIE) index was computed to quantify the method’s ability to recognize active ligands early in the screening process [38,39]. Descriptive statistics, including mean and standard deviation, were applied to analyze docking scores and binding affinities for the screened compounds. For molecular dynamics simulations, RMSD and RMSF profiles were statistically analyzed to assess the stability and flexibility of the ligand–protein complexes over time. All statistical computations were performed using Schrödinger’s Glide module and BIOVIA Discovery Studio 2016 [33].

## 3. Results

### 3.1. Pharmacophore Model Generation

The pharmacophore model developed in this study was derived from the crystal structure of the NA protein from avian influenza H5N1 (PDB ID: 2HTQ) based on the binding interactions of Zanamivir within the active site. The model identified four essential features: one hydrogen bond acceptor, two hydrogen bond donors, and one negatively charged group, reflecting critical motifs necessary for strong and specific binding to neuraminidase. These findings are in agreement with previous studies, including Abu Hammad et al. (2009) [53], who constructed a pharmacophore model from 22 diverse neuraminidase inhibitors incorporating similar interaction features such as hydrogen bonding and charged groups. Similarly, Batool et al. (2016) [1] developed a pharmacophore model for H5N1 neuraminidase featuring comparable interaction motifs, confirming the relevance of these chemical moieties for effective inhibition. Moreover, Rohini and Shanthi (2018) [54] identified similar essential features—hydrogen bond donors and acceptors and negatively charged groups—in their pharmacophore modeling and virtual screening approaches. Collectively, these studies support the validity and relevance of the pharmacophore model proposed in the present work, highlighting the universal importance of these features in the design of neuraminidase inhibitors.

**Figure 2:**
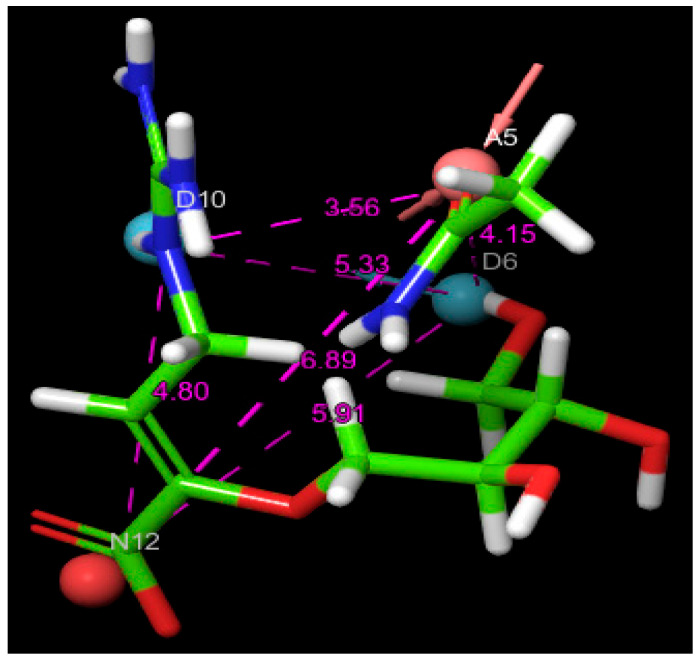
Pharmacophore features generated for Zanamivir with measured inter-feature distances. The color-coded spheres indicate the pharmacophore features: pink represents hydrogen bond acceptors (A5), red denotes negatively charged groups (N12), and blue corresponds to hydrogen bond donors (D6, D10).

Each pharmacophoric element was represented visually in the model, with the hydrogen bond acceptor shown as a pink sphere, the hydrogen bond donors as blue spheres, and the negatively charged group as a red sphere. Figure 2 illustrates the spatial configuration of these features, providing a structural basis for the virtual screening of potential inhibitors from the natural compound library.

### 3.2. Validation of Molecular Docking Protocol

To ensure the reliability of the molecular docking protocol employed in this study, a validation procedure was carried out using enrichment studies. The performance of the protocol was evaluated using statistical metrics such as the Receiver Operating Characteristic (ROC) curve and the Robust Initial Enhancement (RIE) score. The ROC value obtained was 0.92, and the RIE score reached 7.22, both of which indicate strong discriminatory power. A ROC value exceeding 0.7 is generally accepted as a threshold for effective virtual screening and docking performance. Further validation was provided by the SP docking results, which generated an enrichment curve positioned above the diagonal and close to the *X*-axis—corresponding to high sensitivity. This trend reflects a notable enrichment of active compounds, confirming the capability of the docking protocol to prioritize true positives efficiently. In addition, the Area Under the Accumulation Curve (AUC) reached a value of 0.91, which is close to the optimal theoretical performance of 1.0. This result underscores the robustness and reliability of the screening methodology. The use of enrichment metrics such as ROC, RIE, and AUC has become standard in validating docking protocols for virtual screening studies [36]. Supplementary Figure S1 provides a visual representation of the enrichment performance, while detailed numerical values are summarized in Table 1.

### 3.3. Virtual Screening and Docking Studies

With the advancement of computational technologies, virtual screening has become an indispensable component of modern pharmaceutical research. This approach facilitates the identification of bioactive compounds by analyzing molecular structures and predicting their interactions with biological targets [55,56]. In this study, the e-pharmacophore model designated as ADDN was employed to screen a library of 47,781 natural compounds retrieved from the PubChem database. Prior to pharmacophore screening, the compound set was refined using the Canvas Property Filter to ensure compliance with essential drug-like properties. Compounds with a fitness score of 1.5 or higher were selected for further molecular docking analysis to evaluate their potential interaction with the active site of the NA protein. This step significantly enhanced the efficiency and reliability of the virtual screening pipeline. The initial phase of molecular docking involved Glide/SP (Standard Precision) docking of 1829 compounds, which served to eliminate weak binders. Subsequently, the most promising candidates were subjected to Glide/XP (Extra Precision) docking for a more rigorous evaluation of binding affinity and interaction patterns.

From this two-tiered screening process, three natural compounds emerged as top candidates: CID 102209473 (C1), CID 85692821 (C2), and CID 45379525 (C3). These compounds exhibited the highest docking scores, ranging from –10.503 to –9.433 kcal/mol, as detailed in Table 2. A comprehensive overview of the three selected compounds—including their synonyms, IUPAC names, natural sources, chemical families, and relevant bibliographic references—is presented in Table S1.

**Figure 3:**
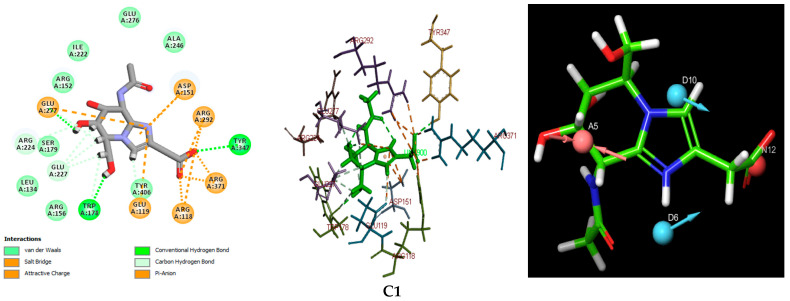

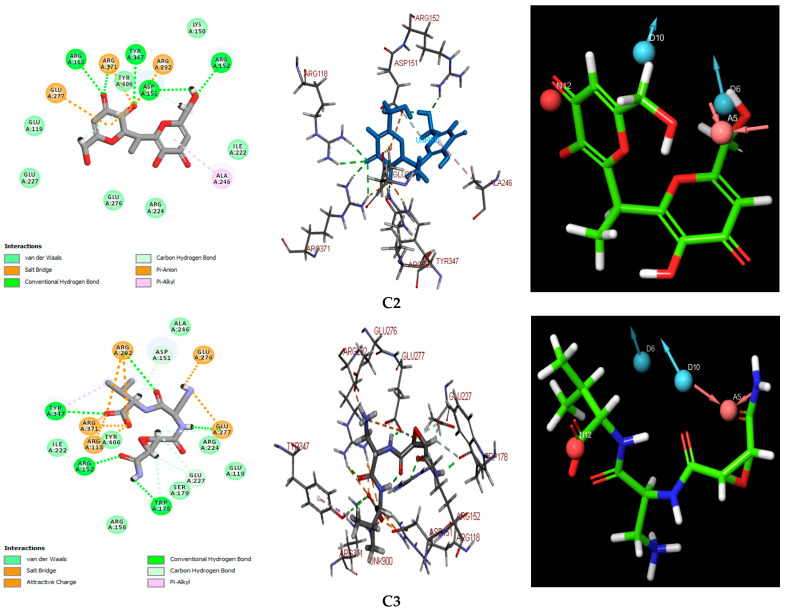
Two-dimensional and three-dimensional images of the docked hit compounds within the active site of the avian influenza H5N1 (PDB ID: 2HTQ) NA protein and the pharmacophoric features predicted for these ligands. Color-coded spheres represent the pharmacophore features: pink for hydrogen bond acceptors, red for negatively charged groups, and blue corresponds to hydrogen bond donors. The dashed lines represent interactions between the ligand and the corresponding amino acid residues.

**Figure 4:**
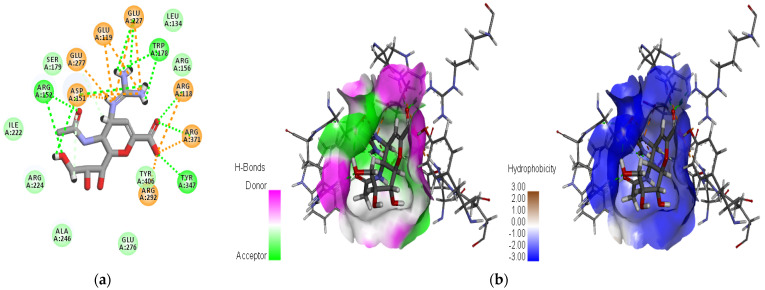
Zanamivir binding position on NA active site: (**a**) 2D representation of ligand–target interactions; (**b**) surface of hydrogen and hydrophobic interactions.

For comparative purposes, two well-established antiviral agents—Zanamivir, used as the reference molecule, and Oseltamivir—were included in this study due to their proven efficacy against influenza viruses. The XP docking scores were calculated to be −10.168 kcal/mol for Zanamivir and −8.848 kcal/mol for Oseltamivir. Notably, compounds C1 and C2 exhibited XP docking scores lower than both Zanamivir and Oseltamivir, indicating stronger predicted binding affinities and suggesting particularly favorable biochemical interactions within the neuraminidase active site. These findings highlight the considerable potential of C1 and C2 as lead candidates for further therapeutic development. Compound C3, while displaying a docking score lower than Oseltamivir, did not outperform Zanamivir but still demonstrated relevant binding characteristics that justify its inclusion among the most promising hits. All three identified compounds fulfilled the four essential pharmacophoric features established by the e-pharmacophore model, as illustrated in Figure 3. This figure also presents a detailed visualization of the ligand–receptor interactions, enabling a comprehensive interpretation of the docking results. For reference, the binding orientation of Zanamivir within the NA active site is depicted in Figure 4, offering structural context for comparison with the novel candidates.

The docking results for the three natural compounds, as well as the two antiviral reference inhibitors, Zanamivir and Oseltamivir, are summarized in Table 3. A comparative analysis of the interaction profiles reveals a striking similarity in the amino acid residues involved in binding between the studied compounds and the reference inhibitors, thus validating the consistency and reliability of our screening approach. A detailed examination of both 2D and 3D interaction maps (presented in Table 3 and Figure 3) identified two primary modes of interaction between the compounds and the active site of the H5N1 NA protein (PDB ID: 2HTQ). These modes include hydrogen bond interactions and electrostatic interactions. Both of these interaction types were also observed with the reference inhibitors Zanamivir and Oseltamivir, confirming their critical role in facilitating the binding of the ligands to the protein active site, as shown in Table 3.

According to the data summarized in Table 3, the selected compounds demonstrate a significantly higher number of hydrogen bond (H-bond) interactions, with bond distances of less than 3.1 Å, similar to the reference inhibitors. Among the amino acid residues involved in H-bond formation, ARG118, ARG292, ARG371, and TYR347 are identified as key residues due to their direct participation in hydrogen bonding across all ligand–protein complexes analyzed. These interactions are pivotal in the stabilization and specificity of ligand–protein binding, as shown in previous studies [57,58,59,60,61].

**Figure 5:**
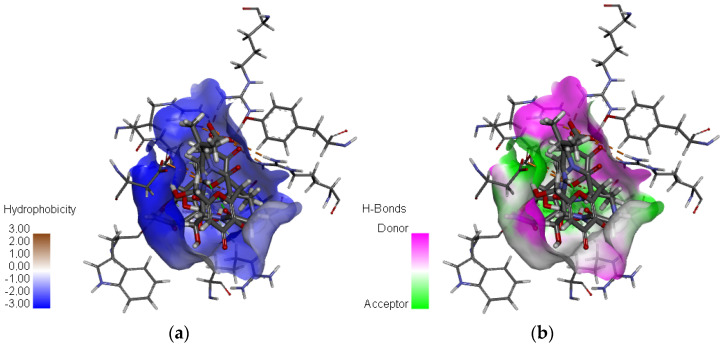
A visualization of the three selected compounds in the active site of the NA protein: (**a**) the surface of hydrophobic interactions; (**b**) the surface of hydrogen interactions.

The results indicate that C1 and C3 form particularly robust biochemical interactions with the target NA protein, marked by a higher number and stability of hydrogen bonds when compared to C2. Additionally, both C1 and C3 exhibit a high number of electrostatic interactions, which are comparable to those observed with Zanamivir. These electrostatic interactions further strengthen the ligand–protein complexes, contributing to their overall binding affinity and specificity [62,63]. Among the three compounds, C3 stands out, with the highest XP docking score of −9.433 kcal/mol, and it also exhibits the highest number of hydrogen bonds (18 in total) and electrostatic interactions (10), suggesting the most favorable overall interaction profile with the target protein. The C2 and C3 compounds each form a single hydrophobic interaction, involving the residues ALA246 and TYR347, respectively. While these interactions are limited in number, they are likely to contribute significantly to the binding energy, especially in localized hydrophobic pockets, as illustrated in Figure 5. These results highlight the complementary role of hydrophobic interactions in stabilizing ligand–protein complexes, particularly when they coexist with more dominant polar and electrostatic interactions [27,34]. The key residues identified in our study—Arg118, Arg152, Glu119, Asp151, Trp178, Ile222, Glu227, Arg292, and Arg371—are consistent with those reported in previous studies on neuraminidase inhibitors [64,65]. This agreement supports the validity of our docking and pharmacophore models in capturing the essential interactions within the active site of the NA protein (PDB ID: 2HTQ).

### 3.4. Prediction of ADMET Profiles

The top leads identified through XP molecular docking, using Schrödinger software, were subjected to comprehensive evaluation in ADMET studies. The selection of promising compounds is not solely based on their binding affinity or the nature of their molecular interactions; it also includes crucial pharmacokinetic and therapeutic potential parameters. These factors are vital for assessing the compounds’ efficacy and safety as potential therapeutic candidates [66]. Table S2 presents the physicochemical and medicinal properties of the compounds under investigation. The results show that the membrane permeability of these molecules is generally limited. Among the compounds, C3 exhibits the highest cell penetration potential, with a TPSA (Topological Polar Surface Area) greater than 150 Å, similar to Zanamivir. In contrast, C1 and C2 have TPSA values ranging from 140 Å to 145 Å, indicating slightly lower cell penetration ability. Notably, all tested compounds, including Zanamivir, have logS values of water solubility within the acceptable range (−4 to 0.5), suggesting adequate aqueous solubility for potential therapeutic use. The logP values for all compounds, including Zanamivir, are negative and fall within the acceptable range (<5), indicating a hydrophilic nature and a low ability to cross the lipid bilayer of cell membranes [67,68]. These findings align well with the TPSA values, further confirming the compounds’ limited membrane permeability.

**Figure 6:**
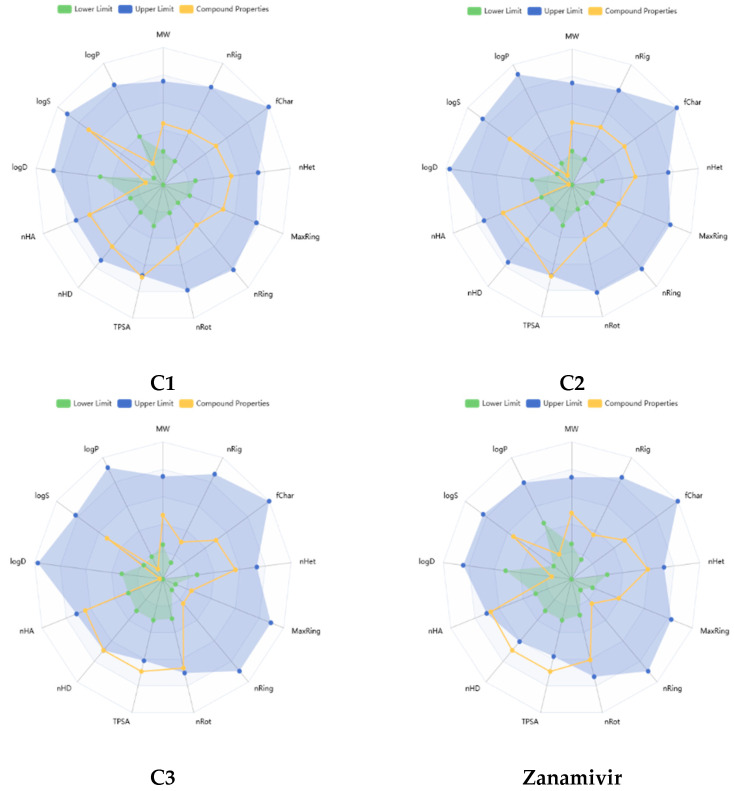
Physicochemical radar chart of top leads and Zanamivir.

Figure 6 presents a radar chart illustrating the physicochemical properties of the top three compounds (C1, C2, and C3) alongside Zanamivir as a comparison, generated using the ADMET Lab 3.0 server. As shown, the properties of C1 and C2 generally fall within the upper limits (highlighted in blue), indicating favorable profiles. C3, while also performing well, slightly exceeds the recommended range for polarity. In contrast, Zanamivir shows a significant deviation, with TPSA > 140 Å and nHD > 5, indicating that its polarity and hydrogen bond donor count are outside the optimal range for drug candidates.

The results indicate that all of the compounds studied are characterized by easy synthesis as their probability values are all lower than six. Additionally, these compounds fulfill the criteria established by several established drug-likeness rules. Specifically, they meet the Lipinski rule of five [69] (MW < 500 D, nHA < 10, nHD < 5, logP < 5), the Pfizer rule [70] (logP < 3, TPSA > 75 Å^2^), the GSK rule [71] (MW < 400 D, logP < 4), and the Golden Triangle rule [72], all of which indicate optimal bioavailability and strong similarity to known drug molecules. Moreover, no warnings were flagged in the Bristol Myers Squibb (BMS) or Pan Assay Interference (PAINS) rules, suggesting that these compounds have a low risk of generating false positives or misleading results. Table 4 presents the predictions for the absorption, distribution, metabolism, excretion, and toxicity (ADMET) properties of the three lead compounds and Zanamivir, as analyzed using the ADMETLab 3 software.

- *Absorption*: Concerning the permeation of compounds through human colon adenocarcinoma cell lines (Caco-2), all studied compounds exhibit values lower than −5.15 log cm/s, indicating a low ability to cross the intestinal barrier, which suggests limited absorption. Compound C2 stands out as an exception, showing higher permeability, potentially enhancing its intestinal absorption. The human intestinal absorption (HIA) of compounds C1 and C2 is limited, with values of 15.2% and 5.0%, respectively. In contrast, compound C3 demonstrates a moderate absorption of 62.2%. Zanamivir shows a high absorption rate of 95.8%, indicating excellent bioavailability and optimal intestinal absorption.
- *Distribution*: The distribution of the compounds was evaluated using key parameters, including blood–brain barrier (BBB) permeability, plasma protein binding (PPB), and the volume of distribution (VD). All compounds, including Zanamivir, exhibit probability values close to zero for BBB permeability, suggesting negligible CNS penetration. Since influenza primarily affects respiratory tissues, this limited penetration into the CNS does not interfere with the drug’s action in the most affected areas, such as the lungs [73]. Regarding PPB, the calculated values for all compounds are below 90%, suggesting a relatively high-unbound fraction in the bloodstream, which may support their availability and therapeutic efficacy. For VD, the predicted values are low (<−0.15), indicating preferential distribution of the compounds in the plasma compartment rather than in tissues.
- *Metabolism and Excretion*: The inhibition of cytochrome P450 enzymes is a key factor in drug metabolism. Table 4 reveals that none of the tested compounds significantly inhibited the major cytochrome P450 enzymes (CYP1A2, CYP2C19, CYP2C9, CYP2D6, amd CYP3A4), with values ranging from 0 to 0.005, suggesting minimal interaction with the relevant metabolic pathways. In terms of clearance, compounds C1, C3, and Zanamivir show low probabilities (less than 5), indicating reduced clearance, while C2 exhibits a moderate rate of renal clearance (9.627). It is worth noting that low clearance is often desirable in drug discovery as it helps reduce the required dosage while increasing drug exposure [74]. All compounds display relatively short half-lives (T1/2), ranging from 1.378 to 2.395 h, indicating rapid elimination from the body. Their limited presence in the biological system underscores their rapid excretion.
- *Toxicity*: The ADMETLab 3 results (Table 4) indicate safe profiles for cardiotoxicity (hERG channel inhibition) and respiratory toxicity. Regarding mutagenicity, the lowest probability values were observed for Zanamivir and C1, with values of 0.209 and 0.394, respectively, indicating a low mutagenic risk for these compounds. In contrast, C2 and C3 display moderate probability values of 0.705 and 0.782, respectively.

Table 5 presents the toxicity predictions from the Protox-II assessment, which evaluates hepatotoxicity, carcinogenicity, mutagenicity, and cytotoxicity. Similar to Zanamivir, the top three leads show favorable safety profiles, with no significant risks for hepatotoxicity, carcinogenicity, mutagenicity, or cytotoxicity. These findings suggest that these compounds pose limited toxicological risks, enhancing their potential for therapeutic or industrial applications.

### 3.5. Frontier Molecular Orbitals Analysis and Global Reactivity Descriptors

The global reactivity descriptors for the three natural compounds studied, namely C1, C2, and C3, were obtained through DFT calculations. The values for each compound, as summarized in Table 6, provide insights into their electronic properties and reactivity. These descriptors are important for understanding the chemical behavior and stability of the compounds, which are crucial for evaluating their potential as therapeutic agents.

**Figure 7:**
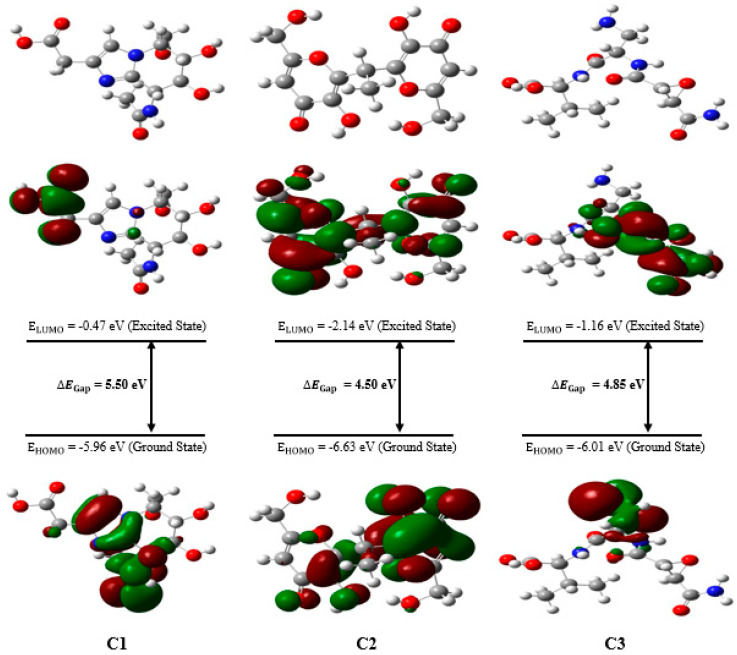
The optimized chemical structures of the compounds analyzed, highlighting the molecular orbitals HOMO and LUMO, as well as their energy gap. The red and green colors indicate the positive and negative phases of the molecular orbitals, respectively.

The results reveal that compound C1 has the highest value of E_HOMO_, suggesting increased reactivity toward electrophiles. On the other hand, compound C2 exhibits the lowest E_LUMO_ value, indicating a greater tendency to interact with nucleophiles. Figure 7 displays the optimized structures of the investigated compounds, highlighting the HOMO and LUMO molecular orbitals, as well as their corresponding energy gaps. In this figure, the red and green colors represent the positive and negative phases of the molecular orbitals, respectively.

**Figure 8:**
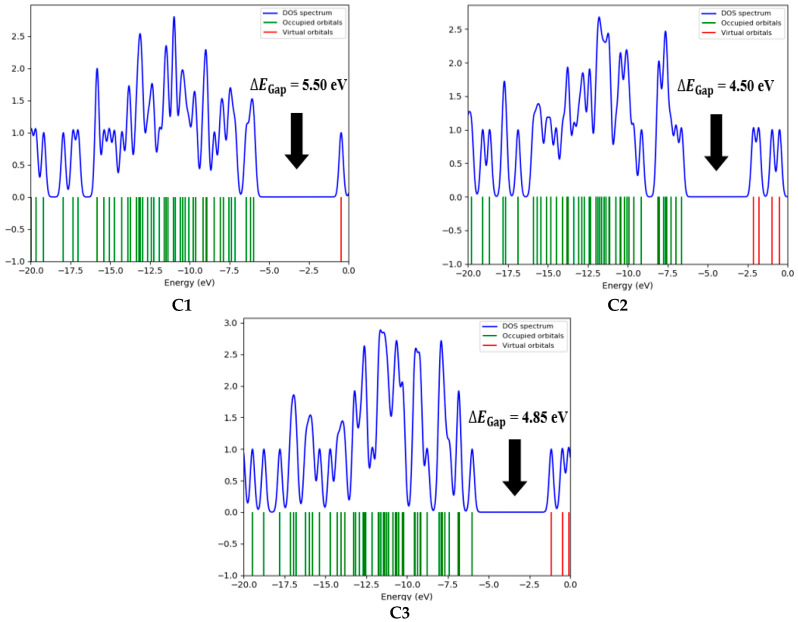
The DOS spectrum for the analyzed compounds.

The energy gap (${∆E}_{Gap}$) is a crucial parameter for predicting the reactivity and chemical stability of a compound. The high value of ${∆E}_{Gap}$ observed for compound C1 indicates greater chemical stability compared to the other compounds studied. Indeed, a larger ${∆E}_{Gap}$ between the boundary molecular orbitals, such as HOMO and LUMO, is typically associated with better thermodynamic and chemical stability as it makes electronic transitions more difficult. In contrast, compounds C2 and C3, which exhibit a smaller energy gap, are expected to have increased reactivity and lower stability. A smaller ${∆E}_{Gap}$ facilitates electronic transitions and interactions with other chemical species, thereby enhancing their chemical reactivity. Figure 8 presents the DOS spectrum generated from the Gauss Sum program for the three natural compounds, confirming the calculated ${∆E}_{Gap}$ values.

The concepts of chemical hardness and softness are fundamental in assessing both the stability and reactivity of molecules. Compound C2, with the lowest chemical hardness (η = 2.25 eV) and the highest chemical softness (S = 0.22 eV), is the most reactive of the compounds studied. Compound C1 is considered the most stable of the three as it has the highest value of chemical hardness (η = 2.75 eV), which also contributes to its greater stability. Chemical potential (μ) corresponds to the tendency of a system to donate electrons, and a negative chemical potential reflects the stability of the complex, meaning it does not spontaneously decompose into its constituents [75]. Table 6 shows that compound C2 has the lowest chemical potential (μ) and the highest electronegativity (χ), suggesting that it functions as a strong electron acceptor. In contrast, compound C1 has the highest chemical potential, implying that it could interact more readily with the environment to exchange electrons.

The electrophilicity index (ω) is a key quantum parameter used to quantify the chemical reactivity of molecules. It represents the ability of a chemical species to accept electrons, evaluating its potential for interaction with biological receptors, particularly in drug design. A low value of ω characterizes a good nucleophile, making it more reactive, while a high value indicates a powerful electrophile [75,76]. Based on the results in Table 6, compound C2 exhibits the highest reactivity, with a ω value greater than 4 eV, followed by compound C3, with an electrophilicity index of 2.65 eV. Compound C1, characterized by a high nucleophilicity index (N) and the lowest value of ω, stands out as the most nucleophilic compound among the studied compounds. Notably, the HOMO energy of the reference system (TCE) was determined to be −9.16 eV using the DFT/B3LYP method with the 6-31G basis set [43].

### 3.6. Molecular Electrostatic Potential (MESP) Analysis

**Figure 9:**
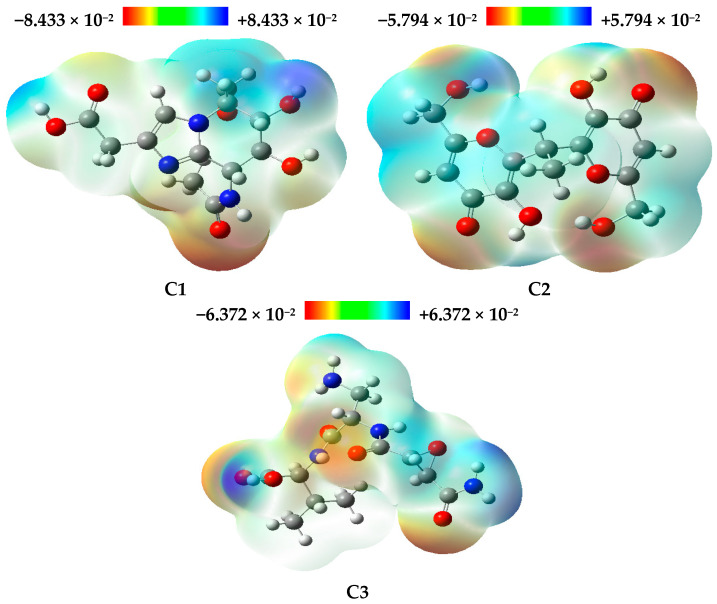
MESP maps of the analyzed compounds with color-coded balls indicating electrostatic potential: red (negative, electrophilic attack sites), blue (positive, nucleophile attack sites), and green (neutral).

The MESP (Molecular Electrostatic Potential) is a critical tool in theoretical chemistry for identifying the reactive sites of organic molecules, particularly those prone to electrophilic and nucleophilic attacks [77]. The MESP map allows for the efficient prediction of molecular interactions based on different geometries. In this study, the MESP of the three natural compounds was calculated by geometric optimization using the B3LYP/6-31G method, as shown in Figure 9. The MESP is an essential parameter for analyzing chemical reactivity as it reveals both the shape and size of a molecule, along with the distribution of its electrostatic potential. Its application is indispensable for understanding the relationships between molecular structure and physicochemical characteristics [78]. The MESP for the three natural compounds was calculated to pinpoint their reactive sites. The areas of maximum negative potential, depicted in red, indicate regions favorable for electrophilic attack, while the areas of maximum positive potential, shown in blue, are indicative of nucleophilic attack sites. Neutral potential regions are represented in green.

### 3.7. MD Simulations

In this study, molecular dynamics (MD) simulations were performed to investigate the structural behavior and interaction profiles of four ligand–protein complexes involving the neuraminidase (NA) enzyme of avian influenza H5N1 (PDB ID: 2HTQ). The tested ligands included three candidate compounds (C1, C2, and C3) and the reference antiviral drug Zanamivir. The resulting complexes—C1-NA, C2-NA, C3-NA, and Zanamivir-NA—were analyzed over a 100 ns simulation period to assess their conformational stability, residue flexibility, and interaction continuity within the active site of the NA protein. The following sections present a comparative analysis of the RMSD, RMSF, and residue–ligand contact maps to highlight the dynamic performance of each complex.

**Figure 10:**
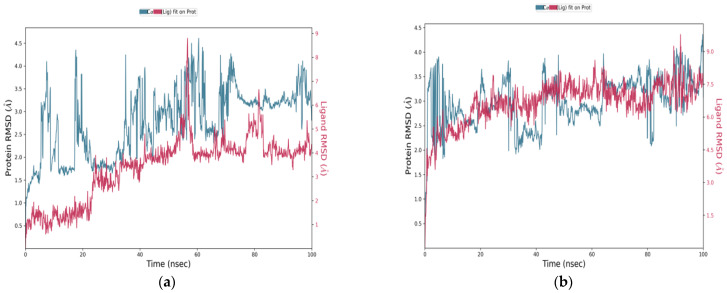

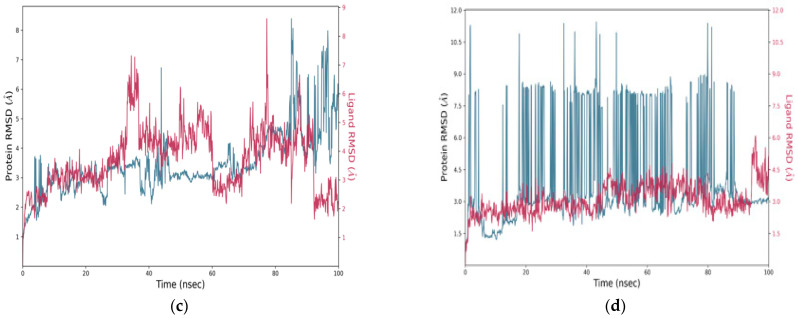
RMSD (root mean square deviation) plots of ligand–NA complexes over 100 ns simulation time. (**a**) C1–NA complex (PDB ID: 2HTQ), (**b**) C2–NA complex (PDB ID: 2HTQ), (**c**) C3–NA complex (PDB ID: 2HTQ), and (**d**) Zanamivir–NA complex (PDB ID: 2HTQ).

The RMSD plots over 100 nanoseconds (Figure 10) reveal significant differences in the behavior of the candidate compounds compared to the reference ligand, Zanamivir. These differences reflect variations in structural stability and anchoring within the active site. The C1-NA complex (Figure 10a) showed an initial fluctuation in the protein’s RMSD, reaching about 2.5 Å, followed by stabilization around 3.0–3.5 Å, indicating structural reorganization followed by a stable phase. The ligand’s RMSD remained generally below 5 Å, suggesting relative stability in the binding site. For the C2-NA complex (Figure 10b), the protein’s RMSD remained in the range of 2.5–3.5 Å without abrupt variations, reflecting higher global stability. The ligand’s RMSD was relatively stable around 6.5–7.5 Å, indicating consistent and maintained positioning throughout the simulation. The C3-NA complex (Figure 10c) showed RMSD fluctuations between 2.5 and 4.0 Å, with a tendency toward stabilization in the second half of the simulation. The ligand maintained an RMSD between 4.5 and 6.5 Å, with a noticeable improvement in the latter phase, indicating progressive adaptation to the active site. In contrast, the Zanamivir-NA complex displayed unstable behavior, with the protein RMSD ranging from 2.0 to 11.0 Å, accompanied by frequent and marked fluctuations (Figure 10d). The ligand RMSD occasionally exceeded 8.0 Å, indicating considerable structural instability and potential partial disengagement from the binding site. These results suggest that C1 and C2, in particular, exhibit superior dynamic stability compared to the reference molecule, reinforcing their potential as promising therapeutic candidates.

To complement the RMSD analysis and gain deeper insights into the flexibility and interaction patterns of the ligand–protein complexes, further investigations were carried out through root mean square fluctuation (RMSF) profiles and interaction mapping.

**Figure 11:**
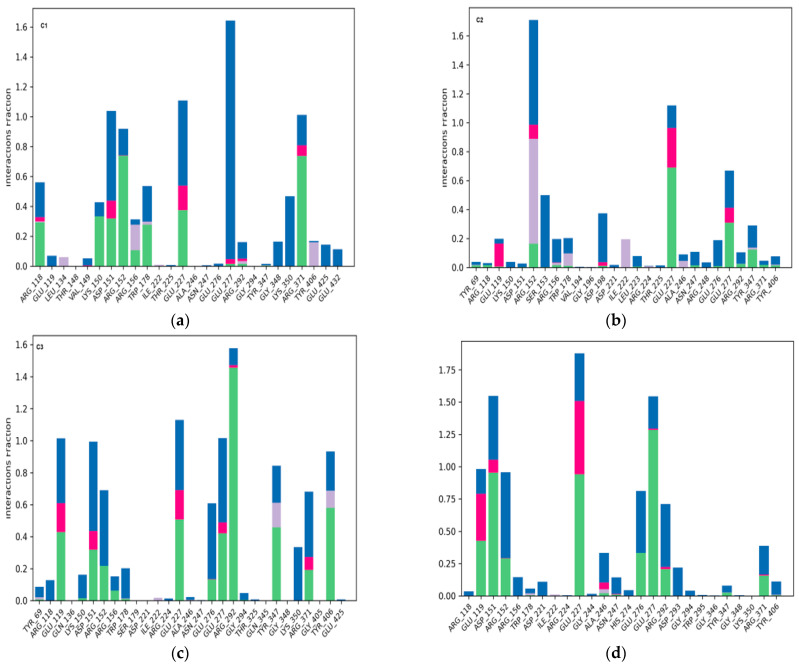
NA interactions with ligands over 100 ns simulation time, with color-coded interaction types: green (hydrogen bonds), purple (hydrophobic interactions), pink (ionic interactions), and blue (water bridges). (**a**) C1–NA complex (PDB ID: 2HTQ), (**b**) C2–NA complex (PDB ID: 2HTQ), (**c**) C3–NA complex (PDB ID: 2HTQ), and (**d**) Zanamivir–NA complex (PDB ID: 2HTQ).

C1 formed a diverse array of interactions distributed across several active site residues (Figure 11a). These included strong water bridges, notably with GLU277 (interaction fraction > 1.6) and with ARG152, ARG118, GLU119, and ASP151 (values between 0.5 and 1.1). Clear ionic bonds were also observed, particularly with GLU277, GLU119, and ARG371, indicating solid electrostatic interactions between oppositely charged groups. Furthermore, the ligand formed notable hydrogen bonds with ASP151, ARG152, LYS151, and ARG371. Hydrophobic interactions, though present, were limited and involved primarily ARG156, LEU134, and TYR406 at relatively low frequency. This interaction profile reflects an effective three-dimensional anchoring of the ligand in the active site, supported by a combination of water bridges and ionic interactions. This aligns well with the low RMSD of the ligand (<5 Å) and the protein complex stability (3–3.5 Å), corroborated by high docking performance.

C2 established specific interactions with several key residues in the active site (Figure 11b). Significant ionic bonds were formed, particularly with GLU227, GLU119, and ARG151, although frequencies varied. Clear hydrogen bonds were observed with TYR347 and GLU277, indicating directional anchoring in the catalytic site. Additionally, water bridges were detected between the ligand and ASP198 and ARG152, with an interaction frequency around 0.6, further strengthening complex stability. Hydrophobic interactions involving ILE222, TRP178, and ARG152 suggested ligand insertion into a nonpolar region of the active site. Despite a relatively high ligand RMSD (6.5–7.5 Å), the combination of electrostatic and hydrophobic interactions allowed for prolonged and relatively stable ligand retention. This stability may be attributed to a balanced interaction profile between polarity and flexibility, explaining the ligand’s persistence in the active site throughout the simulation.

C3 showed a particularly rich and diverse interaction profile (Figure 11c). It formed strong hydrogen bonds, notably with ARG292 (interaction frequency > 1.4), and important water bridges with essential residues such as GLU277, ASP151, ARG152, GLU119, and ARG371, reinforcing ligand integration into the active site. Ionic bonds were well represented, involving GLU227, ASP151, GLU119, and ARG371, supporting effective electrostatic stabilization. Additionally, notable hydrophobic interactions with TYR347 and TYR406 suggested favorable positioning in nonpolar binding pockets. This diversity of interactions highlights C3′s ability to simultaneously exploit different molecular forces to ensure robust binding within the active site. This is consistent with a moderate ligand RMSD (4.5–6.5 Å) and a notable improvement in structural stability in the second half of the simulation, indicating good adaptive flexibility of the ligand–protein complex.

The reference compound Zanamivir initially exhibited strong interactions with several key residues in the active site (Figure 11d). It formed marked hydrogen bonds with GLU277 (interaction frequency ~1.3), as well as with GLU119, ASP151, and ARG152 (0.5–1.0 range). It also showed notable reliance on ionic interactions, mainly with ARG152 and GLU277, with minor contributions from GLU119 and ASP151. Water bridges were also observed, particularly with ARG152, GLU277, ARG292, and GLU119, reflecting water involvement in complex stabilization. However, there was a near absence of hydrophobic interactions, limiting the ligand’s overall stability in a dynamic environment. This imbalance in interaction types may explain the marked fluctuations observed in the molecular simulation, where the protein RMSD exceeded 10 Å and the ligand RMSD surpassed 8 Å. Despite strong initial affinity, Zanamivir exhibited significant long-term structural instability, likely due to a lack of complementarity with the nonpolar regions of the active site.

**Figure 12:**
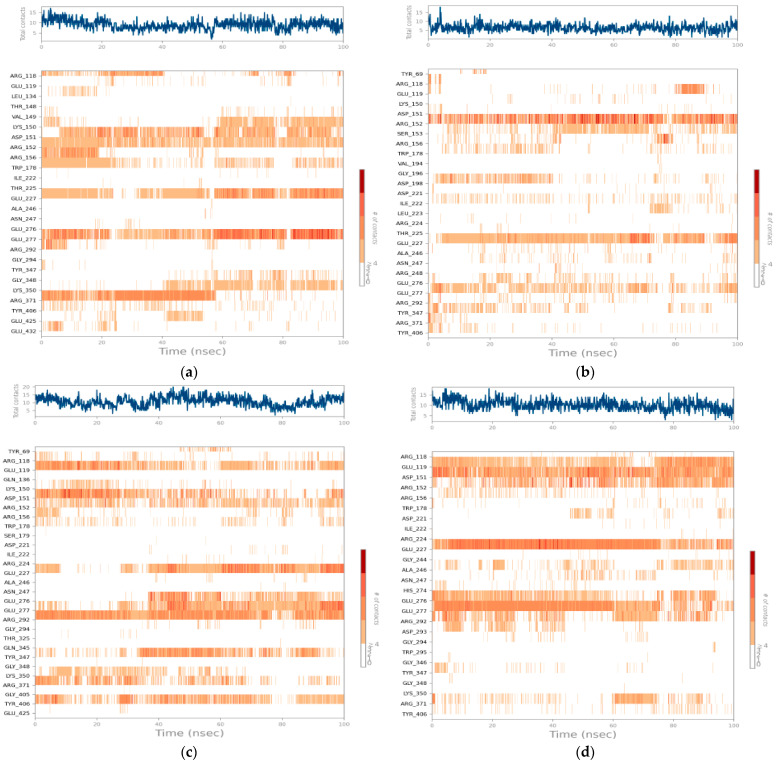
Residue–ligand contact maps at 100 ns for NA complexes. The top panel shows the total number of hydrogen bonds, hydrophobic, ionic, and water bridge interactions over time. The bottom panel details residue-specific contacts per simulation frame, with darker orange indicating multiple simultaneous interactions. (**a**) C1–NA complex (PDB ID: 2HTQ), (**b**) C2–NA complex (PDB ID: 2HTQ), (**c**) C3–NA complex (PDB ID: 2HTQ), and (**d**) Zanamivir–NA complex (PDB ID: 2HTQ).

An analysis of the contact maps during the 100-nanosecond molecular simulation (Figure 12) revealed notable differences in ligand behavior in terms of stability and the persistence of interactions with active site residues.

Compound C1 (Figure 12a) exhibits a high and relatively constant interaction density, fluctuating between 10 and 15 contacts throughout the trajectory. This profile reflects remarkable stability of the complex, with residues such as ARG224 and GLU277 maintaining long-lasting contacts throughout the simulation. Sustained interactions are also observed with ARG152, ASP151, and ARG292, reinforcing the idea of deep, multiregional ligand anchoring. The consistency in these interactions supports the low RMSD values and confirms the stability of the complex over time.

Compound C2 (Figure 12b) displays more fluctuating dynamics, with a total number of contacts ranging from 5 to 12. The most frequent interactions involve ARG151, SER153, GLU277, and ALA246, but these interactions are more intermittent and less persistent over time. This partial instability could explain the increased mobility of the ligand in the active site, as revealed by the higher RMSD values observed previously. The fluctuation in the number of contacts suggests that C2 is not as firmly anchored in the active site as C1, leading to greater mobility and reduced stability.

Compound C3 (Figure 12c), on the other hand, demonstrates intermediate but very promising behavior. The number of contacts fluctuates, sometimes reaching nearly 18, with a strong intensity in the second half of the simulation. C3 establishes consistent interactions with key residues, including GLU277, ARG292, GLU119, and ARG371. This prolonged bond retention, illustrated by strong horizontal bands on the map, demonstrates excellent structural complementarity and a good dynamic fit within the enzymatic site. The high number of contacts, particularly in the latter half of the simulation, highlights C3’s ability to stabilize itself through consistent and diverse interactions.

Zanamivir (Figure 12d), in contrast, initially displayed a numerically similar interaction profile to C1, but a detailed analysis of the interaction map revealed a lack of continuity. Frequent breaks in interactions, even at critical residues such as ARG152, GLU277, and ARG224, suggest gradual destabilization over time. This lack of interaction stability explains the large variations in dynamic parameters such as the RMSD and RMSF despite its initial binding energy being comparable to that of the candidate compounds.

In contrast to Zanamivir, complexes C1-NA, C2-NA, and C3-NA exhibited better dynamic stability. The C1-NA complex is characterized by rich, balanced interactions and a low ligand RMSD deviation, confirming its structural stability throughout the simulation. The C2-NA complex maintained its stability despite greater mobility, thanks to a combination of ionic and hydrophobic interactions. The C3-NA complex, incorporating a variety of ligand types, adapted effectively within the active site, showcasing excellent flexibility and stability in the latter part of the simulation.

Overall, the candidate compounds C1, C2, and C3 displayed better dynamic stability compared to Zanamivir, which suffered from a lack of interaction diversity, particularly the absence of hydrophobic bonds. This imbalance likely contributed to Zanamivir’s lower stability over time despite its initial strong binding.

## 4. Discussion

The H5N1 avian influenza virus continues to pose a serious threat to human health due to its high virulence and ability to infect multiple species. Although some NA inhibitors are already in clinical use, their efficacy is declining in the face of the emergence of resistant strains, highlighting the urgent need for new and better treatments. In this context, NA remains a priority therapeutic target. The development of in silico methods, such as virtual screening, molecular modeling, and the dynamics of ligand–protein interactions, is now accelerating the search for new active molecules with high inhibitory potential. These tools offer a promising way to identify innovative candidates capable of overcoming the current limitations of available treatments. The aim of the present study was therefore to use in silico approaches to identify new potential inhibitors of influenza virus NA. The results obtained through virtual screening and molecular docking indicate the promising activity of compounds C1, C2, and C3 as potential inhibitors of the neuraminidase (NA) enzyme of the H5N1 virus. An electronic pharmacophore (ADDN) model was constructed based on the steric and electrostatic properties extracted from the reference ligand (Zanamivir), enabling the identification of the essential structural features required for effective binding to the enzyme’s active pocket.

The high performance of compounds C1 and C2 in the SP and XP docking models demonstrates strong affinity for the NA active site, exceeding those of known antivirals (Zanamivir and Oseltamivir). This indicates that these two compounds possess chemical structures capable of achieving energy stability within the active site thanks to their structural conformation and electronic configuration that fit the environment surrounding the pocket. A detailed analysis of the interaction patterns revealed that C1 and C2 form direct hydrogen bonds with the key residues ARG118, ARG292, ARG371, and TYR347, which are conserved residues known to play a pivotal role in the NA catalytic mechanism. These bonds mimic those formed by Zanamivir, indicating that the two compounds not only occupy the active site but also contribute to enzyme inhibition by disrupting the active structure essential for its function. Furthermore, the observed electrostatic interactions contribute to enhancing the stability of the molecular complex, which is critical for maintaining inhibition efficacy during biological dynamics. Compound C3, despite its relatively lower binding score, exhibits a remarkably rich interaction profile. It forms a total of 18 hydrogen bonds and 10 electrostatic interactions, high numbers that indicate multiple anchoring points between the compound and the enzyme, potentially enhancing its long-term stability within the active pocket. This multipoint interaction pattern can compensate for the initial weak binding energy and give the compound the ability to resist minor structural changes in the enzyme, as expected in mutant strains. Taken together, these results indicate that the three molecules interact with NA via mechanisms similar to reference drugs, offering additional interaction diversity and structural fitness, supporting the validity of the ADDN model as an effective virtual screening tool. The results are consistent with previous reports on the development of novel NA inhibitors in the context of strain resistance [1,27,79,80]. Pharmacokinetic and Toxicological Evaluation (ADMET) is a pivotal stage in the drug discovery process, used to determine whether a compound is a viable and safe therapeutic option. In this study, the results reveal that the three main compounds (C1, C2, and C3) exhibit very promising ADMET properties, enhancing their potential as potential therapeutic candidates. First, the high TPSA (Topological Polar Surface Area) value and negative logP value indicate relatively limited membrane permeability, a factor associated with reduced absorption of the compound across biological barriers, particularly the blood–brain barrier (BBB). This is an advantage in the context of treating respiratory viruses, such as H5N1, as the central nervous system is not targeted, reducing the potential for unwanted neurological side effects and enhancing therapeutic concentration in the target tissues. Second, the compounds demonstrated excellent solubility in aqueous media, a key indicator of high bioavailability upon oral administration. Their compliance with a set of well-known pharmacokinetic rules, such as the Lipinski, Pfizer, Golden Triangle, and GSK rules, reinforces the hypothesis that these compounds have properties similar to currently approved drugs whether in terms of absorption, distribution, or stability. Third, the compounds’ metabolic profiles are remarkably favorable as the compounds did not show significant inhibition of cytochrome P450 enzymes, which reduces the risk of drug interactions and enhances their safety in patients taking other medications. Moderate clearance and rapid excretion also indicate a consistent and low-accumulation drug cycle, reducing the potential for chronic cumulative toxicity. Finally, the absence of evidence of hepatotoxicity, cytotoxicity, or mutagenicity—toxicity parameters that typically lead to the failure of drug candidates—represents a key strength. These results not only demonstrate the compounds’ safety but also highlight their structural flexibility and the potential for future improvement without compromising their overall safety profile. The molecular dynamics simulation results indicate that the candidate compounds (C1, C2, and C3) are superior in terms of structural interaction stability with the NA enzyme active site compared to the reference ligand, Zanamivir. For C1-NA, the low root mean square deviation (RMSD) of the ligand reflected strong structural stability throughout the simulation, maintaining a rich and balanced interaction pattern comprising hydrogen bonds and electrostatic interactions. This low RMSD fluctuation indicates that C1 maintained its original position within the active pocket without significant dissociation or sliding, enhancing the persistence of enzyme inhibition over time. 

The elevated RMSD values observed for the C2 and C3 complexes during molecular dynamics simulations can be attributed to the intrinsic flexibility of the ligands and the dynamic nature of their interactions within the neuraminidase active site. For C2, the protein RMSD fluctuated between 2.5 and 3.5 Å, while the ligand RMSD consistently ranged between 6.5 and 7.5 Å, suggesting a degree of flexibility in the ligand’s conformation while maintaining its binding pose. In the case of C3, the observed RMSD fluctuations between 2.5 and 4.0 Å reflect initial structural adaptations, with subsequent stabilization indicative of the system reaching equilibrium. This behavior is characteristic of ligands that undergo conformational adjustments to optimize their interactions within a flexible binding environment. Such dynamics highlight the potential for these ligands to exhibit enhanced adaptability and stable binding in complex biological systems. 

C2-NA, on the other hand, exhibited a relatively higher degree of molecular mobility (higher flexibility), which may initially appear undesirable. However, this mobility proved to be controlled and accompanied by a strong network of ionic and hydrophobic interactions, which maintained the overall stability of the compound within the active pocket. This property gives C2 a dynamic advantage, enabling it to better adapt to minor structural changes in the enzyme resulting from mutations or biological stress. Concerning the C3-NA complex, it exhibited a highly diverse interaction structure, enabling it to gradually adapt to the active pocket environment. Although some structural fluctuations occurred at the beginning of the simulation, the complex stabilized strongly during the latter part of the simulation, indicating a high ability to achieve static–dynamic equilibrium after a period of molecular reorganization.

In contrast, Zanamivir demonstrated good initial stability but rapidly began to lose this stability over time, coinciding with the absence of important hydrophobic bonds within the active pocket. In complex, aqueous enzyme environments, hydrophobic interactions play a crucial role in enhancing the stability of the complex by repelling unwanted water molecules and ensuring the ligand’s containment within the active site. The lack of this type of interaction in Zanamivir may have contributed to the gradual loss of dynamic stability despite its initial strong binding, thus compromising its effectiveness in volatile biological environments.

Thus, these results demonstrate that the richness of interactions (hydrogen, electrostatic, and hydrophobic) is not only key to improving the initial binding energy but is also essential to ensure the stability of the bond over time, which is a prerequisite for inhibitor efficacy in a living biological system. The results obtained not only confirm the relevance of the in-silico screening approach for the discovery of new inhibitors but are also consistent with previous work that has highlighted the limitations of some classical antivirals in a dynamic context [4,81]. In particular, the richness and diversity of interactions observed for C1 and C3, as well as the prolonged stability of C2 despite increased relative mobility, suggest better structural complementarity with the target protein [82]. These observations show promising prospects for the development of new therapeutic candidates with high affinity and stability, justifying further experimental investigations.

## 5. Conclusions

In summary, this study employed an integrated molecular modeling approach, combining e-pharmacophore design, molecular docking, ADMET predictions, density functional theory (DFT), and molecular dynamics simulations, applied to a dataset of 47,781 natural compounds from the PubChem database. The primary aim was to theoretically identify promising antiviral inhibitors targeting the influenza virus. The pharmacophoric model generated (ADDN), which incorporates a hydrogen-bonding acceptor, two donors, and a negatively charged group, successfully selected three compounds (C1: CID 102209473, C2: CID 85692821, and C3: CID 45379525) with high binding affinities and strong interaction profiles with the viral NA target.

The reliability of the molecular docking protocol was reinforced by enrichment calculations, which validated the robustness of the obtained results. Furthermore, the ADMET profiles of the selected compounds indicated favorable bioavailability and low predicted toxicity, making them promising candidates for therapeutic development. DFT calculations highlighted favorable electronic properties in compounds C2 and C3, particularly related to their capacity for effective interactions with viral neuraminidase, thus suggesting their potential in the design of new antiviral agents.

The molecular dynamics simulation results revealed that the C1-NA, C2-NA, and C3-NA complexes exhibited superior dynamic stability, supported by rich and complementary molecular interactions. In contrast, Zanamivir showed reduced stability due to a less diverse interaction profile, particularly lacking hydrophobic contacts, which resulted in higher instability.

Given that this study is primarily based on computational approaches, it is crucial to conduct additional experimental investigations, including in vitro and in vivo studies, to evaluate the safety and efficacy of the identified neuraminidase (NA) inhibitors against the avian influenza virus.

## Figures and Tables

**Table 1:** Performance evaluation of SP molecular docking results using enrichment metrics for validation.

ROC	RIE	AUC	Enrichment Metric	Value
0.92	7.22	0.91	BEDROC (α = 160.90)	0.602
			BEDROC (α = 20.00)	0.468
			BEDROC (α = 8.0)	0.630

**Table 2:** XP docking scores for selected ligands, obtained by GLIDE.

Comp.	XP Score Kcal/mol	Glide Module
CID 102209473	−10.503	−66.663
CID 85692821	−10.281	−45.269
CID 45379525	−9.433	−73.647
Zanamivir	−10.168	−59.276
Oseltamivir	−8.848	−77.308

**Table 3:** The characterization of interactions and molecular bonds of the three compounds identified and the two reference inhibitors with the avian influenza H5N1 (PDB ID: 2HTQ) NA protein.

Comp.	H-Bond		Distance	Number	Hydrophobic	Number	Electrostatic		Number
C1	ARG118	ARG292							
	ARG371	TYR347					ARG118	ARG292	
	GLU277	TRP178	[1.60–3.06]	16	/	/	ARG371	GLU119	12
	ARG224	GLU227					GLU277	ASP151	
	GLU119								
C2	ARG292	ARG371					ARG292	ARG371	4
	ARG118	ARG152	[1.56–2.82]	11	ALA246	1	ASP151	GLU277	
	TYR347	ASP151							
C3	ARG118	ARG292							
	ARG371	GLU276					ARG118	ARG292	
	ARG152	TYR347	[1.44–3.03]	18	TYR347	1	ARG371	GLU276	11
	GLU277	TRP178					GLU277		
	GLU227	ASP151							
Zanamivir	ARG292	ARG371					ARG292	ARG371	
	ASP151	ARG118					ASP151	ARG118	
	ARG152	TYR347	[1.79–2.88]	19	/	/	GLU119	GLU227	11
	TRP178	GLU227					GLU277		
	ASP151								
Oseltamivir	GLU119	ASP151			ALA246		GLU119	ASP151	
	ARG152	ARG292	[1.52–2.56]	9	ILE222	2	GLU227		4
	ARG371								

**Table 4:** ADMET properties of the top leads and Zanamivir.

Category	Property (Unit)	C1	C2	C3	Zanamivir
Absorption	Caco-2 permeability (cm/s)	−6.096	−5.058	−6.126	−5.96
	HIA	0.152	0.05	0.622	0.958
Distribution	BBB penetration (cm/s)	0.0	0.001	0.0	0.001
	PPB (%)	31.596	75.563	18.749	9.564
	VD (L/Kg)	−0.592	−0.164	−0.547	−0.644
Metabolism	CYP1A2 inhibitor	0.0	0.002	0.0	0.0
	CYP2C19 inhibitor	0.0	0.0	0.0	0.0
	CYP2C9 inhibitor	0.0	0.0	0.0	0.0
	CYP2D6 inhibitor	0.0	0.0	0.0	0.0
	CYP3A4 inhibitor	0.0	0.005	0.0	0.0
Excretion	CL (ml/min/Kg)	1.823	9.627	2.361	1.05
	T1/2 (H)	2.395	1.641	1.378	1.876
Toxicity	hERG blockers	0.007	0.021	0.022	0.016
	AMES toxicity	0.394	0.705	0.782	0.209
	Respiratory toxicity	0.081	0.252	0.45	0.143

**Table 5:** Predictions about the toxicity of the top leads and Zanamivir using the Protox-II platform.

Comp.	C1	C2	C3	Zanamivir
Hepatotoxicity	Inactive	Inactive	Inactive	Inactive
Carcinogenicity	Inactive	Inactive	Inactive	Inactive
Immunotoxicity	Inactive	Inactive	Inactive	Inactive
Mutagenicity	Inactive	Inactive	Inactive	Inactive
Cytotoxicity	Inactive	Inactive	Inactive	Inactive

**Table 6:** The DFT calculated global reactivity descriptors (eV) for the selected compounds.

Comp.	E_HOMO_	E_LUMO_	${∆E}_{Gap}$	η	S	μ	χ	ω	N
C1	−5.96	−0.47	5.50	2.75	0.18	−3.21	3.21	1.88	3.20
C2	−6.63	−2.14	4.50	2.25	0.22	−4.39	4.39	4.28	2.53
C3	−6.01	−1.16	4.85	2.42	0.21	−3.59	3.59	2.65	3.15

## Data Availability

All data are contained within the article.

## Supplementary Materials

The following supporting information can be downloaded at: https://www.mdpi.com/article/10.3390/bioengineering12060622/s1, Figure S1: ROC curve after SP docking process; Table S1: Synonyms, IUPAC names, natural sources, and chemical families of the three identified natural compounds; Table S2: Physicochemical and medicinal properties predictions using ADMETLab 3.

## Abbreviations

The following abbreviations are used in this manuscript:

BEDROC	Boltzmann-enhanced discrimination of receiver operating characteristic
2D-QSAR	Two-dimensional quantitative structure–activity relationship
3D-QSAR	Three-dimensional quantitative structure–activity relationship
ADMET	Absorption, distribution, metabolism, excretion, and toxicity
Caco-2	Colon adenocarcinoma cell lines
PAINS	Pan assay interference
XlogP	Octanol–water partition coefficient
MMFF+	Molecular mechanics force field
HOMO	Highest occupied molecular orbital
LUMO	Lowest unoccupied molecular orbital
MESP	Molecular electrostatic potential
RMSD	Root mean square deviation
RMSF	Root mean square fluctuation
SBVS	Structure-based virtual screening
TPSA	Topological polar surface area
AM1	Austin Model 1
AUC	Area under accumulation curve
BBB	Blood–brain barrier
BMS	Bristol Myers Squibb
CNS	Central nervous system
DFT	Density functional theory
HBA	Number of hydrogen bond acceptors
HBD	Number of hydrogen bond donors
HIA	Human intestinal absorption
MTK	Martyna–Tuckerman–Klein
PME	Particle mesh Ewald
PPB	Plasma protein binding
RIE	Robust initial enrichment
ROC	Receiver operating characteristic
SID	Simulation interaction diagram
SPC	Simple point charge
TCE	Tetracyanoethylene
HA	Hemagglutinin
MD	Molecular dynamics simulations
MW	Molecular weight
NA	Neuraminidase
SP	Standard precision
VD	Volume of distribution
XP	Extra precision
A	Hydrogen bond acceptor
D	Hydrogen bond donor
H	Hydrophobic region
N	Negatively charged group
P	Positively charged group
R	Aromatic ring
S	Softness
