# Supplementary material for: Discovery of Novel Natural Inhibitors of H5N1 Neuraminidase Using Integrated Molecular Modeling and ADMET Prediction
Source: Bioengineering (Basel). 2025 Jun 7;12(6):622. doi: 10.3390/bioengineering12060622 (PMC12189692; doi:10.3390/bioengineering12060622)
Supplement: Supplementary file 1 [file bioengineering-12-00622-s001.zip › bioengineering-3648195-supplementary.pdf]

Article

Discovery of Novel Natural Inhibitors of H5N1 Neuraminidase Using Integrated Molecular Modeling and ADMET Prediction

Afaf Zekri [1], Mebarka Ouassaf [1,*], Shafi Ullah Khan [2,3], Kannan R. R. Rengasamy [4,5] and Bader Y. Alhatlani [6,*]

1 Group of Computational and Medicinal Chemistry, LMCE Laboratory, University of Biskra, BP 145, Biskra 07000, Algeria; afaf.zekri@univ-biskra.dz
2 Normandie Univ, Université de Caen Normandie, Inserm U1086 ANTICIPE (Interdisciplinary Research Unit for Cancer Prevention and Treatment), 14000 Caen, France; shafiullahpharmd@gmail.com
3 Comprehensive Cancer Center François Baclesse, UNICANCER, 14076 Caen, France
4 Laboratory of Natural Products and Medicinal Chemistry (LNPMC), Saveetha Medicinal College and Hospitals, Saveetha Institute of Medical and Technical Sciences (SIMATS), Thandalam, Chennai 602105, India; kannan@LNPMC.in
5 Centre of Excellence for Pharmaceutical Sciences, North-West University, Potchefstroom 2520, South Africa
6 Unit of Scientific Research, Applied College, Qassim University, Buraydah 52571, Saudi Arabia
* Correspondence: nouassaf@univ-biskra.dz (M.O.); balhatlani@qu.edu.sa (B.Y.A.)

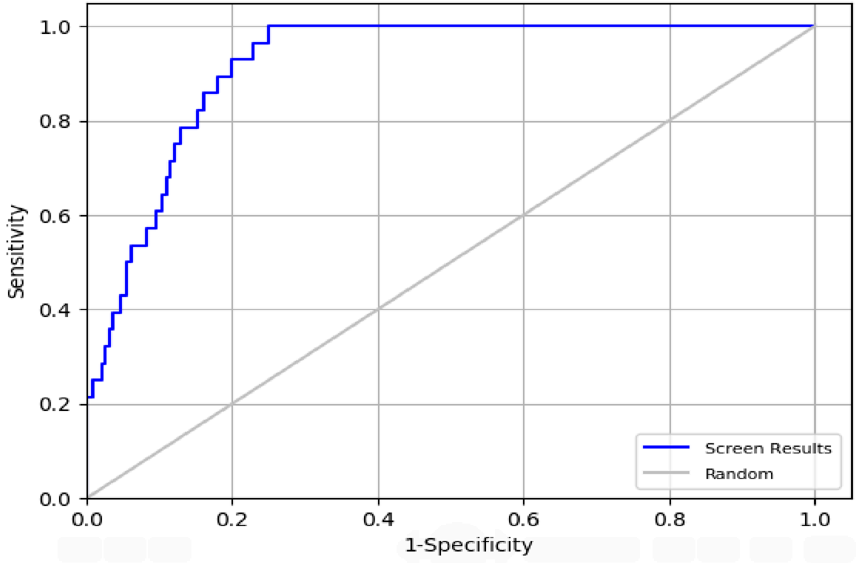

Figure S1. ROC curve after SP docking process.

Table S1. Synonyms, IUPAC names, natural sources, chemical families, and bibliographic references of the three identified natural compounds.

Comp.	Synonym	IUPAC Name	Natural source	Family
C1	Nagstatin	2-[(5R,6R,7S,8S)-8-acetamido 6,7-dihydroxy-5-(hydroxymethyl)-5,6,7,8-tetrahydroimidazo[1,2-a]pyridin-2-yl]acetic acid	Streptomyces amakusaensis MG846-fF3.	Imidazopyridine

C2	Dikojiacid B	3-hydroxy-2-[1-[3-hydroxy-6-(hydroxymethyl)-4-oxopyran-2-yl]ethyl]-6-(hydroxymethyl)pyran-4-one	Aspergillus flavus from the fruit of Garcinia. multiflora	Kojic acid dimer
C3	Dapdiamide E	(2S)-2-[[(2S)-3-amino-2-[(3-carbamoyloxirane-2-carbonyl)amino]propanoyl]amino]-3-methylbutanoic acid	Pantoea agglomerans	Dapdiamide

Table S2. Physicochemical and medicinal properties predictions using ADMETLab 3.

**Comp.**	**C1**	**C2**	**C3**	**Zanamivir**
MW	299.11	310.07	316.14	332.13
Volume	274.181	288.09	293.734	302.525
nHA	9.0	8.0	10.0	11.0
nHD	5.0	4.0	7.0	9.0
TPSA	144.91	141.34	177.14	198.22
logS	-0.612	-1.135	-1.573	-1.429
logP	-1.56	-0.405	-0.421	-2.316
SAscore	4.0	3.0	3.0	4.0
Lipinski Rule	0	0	0	1.0
Pfizer Rule	0	0	0	0
GSK Rule	0	0	0	0
Golden Triangle	0	0	0	0
PAINS	0	0	0	0
BMS	0	0	0	0
